# Supplementary material for: Correlation of Serum N-Acetylneuraminic Acid with the Risk of Moyamoya Disease
Source: Brain Sci. 2023 Jun 5;13(6):913. doi: 10.3390/brainsci13060913 (PMC10296217; doi:10.3390/brainsci13060913)
Supplement: Supplementary file 1 [file brainsci-13-00913-s001.zip › brainsci-2387831-supplementary.pdf]

Article

Correlation of Serum N-Acetylneuraminic Acid with the Risk of Moyamoya Disease

Chenglong Liu [1,2,3,4,5,†], Peicong Ge [1,2,3,4,5,†], Chaofan Zeng [1,2,3,4,5], Xiaofan Yu [1,2,3,4,5], Yuanren Zhai [1,2,3,4,5], Wei Liu [1,2,3,4,5], Qiheng He [1,2,3,4,5], Junsheng Li [1,2,3,4,5], Xingju Liu [1,2,3,4,5], Jia Wang [1,2,3,4,5], Xun Ye [1,2,3,4,5], Qian Zhang [1,2,3,4,5], Rong Wang [1,2,3,4,5], Yan Zhang [1,2,3,4,5], Jizong Zhao [1,2,3,4,5,*] and Dong Zhang [1,2,3,4,5,6,*]

1 Department of Neurosurgery, Beijing Tiantan Hospital, Capital Medical University, 119 South Fourth Ring West Road, Fengtai District, Beijing 100070, China; 18976866841@163.com (C.L.); gepeicong@163.com (P.G.); zchf723@163.com (C.Z.); xfyumail@gmail.com (X.Y.); 18610638233@163.com (Y.Z.); liuwei980227@163.com (W.L.); heqiheng96@mail.ccmu.edu.cn (Q.H.); lijunsheng723@163.com (J.L.); liuxingju006@163.com (X.L.); wangjia1118@hotmail.com (J.W.); yexun@bjtth.org (X.Y.); zhangqianchina@yahoo.com (Q.Z.); ronger090614@126.com (R.W.); yanzhang135@163.com (Y.Z.)

2 China National Clinical Research Center for Neurological Diseases, Beijing 100070, China

3 Center of Stroke, Beijing Institute for Brain Disorders, Beijing 100070, China

4 Beijing Key Laboratory of Translational Medicine for Cerebrovascular Disease, Beijing 100070, China

5 Beijing Translational Engineering Center for 3D Printer in Clinical Neuroscience, Beijing 100070, China

6 Department of Neurosurgery, Beijing Hospital, Beijing 100730, China

* Correspondence: zhaojizong@bjtth.org (J.Z.); zhangdong0660@aliyun.com (D.Z.)

† These authors contributed equally to this work.

Table S1. Independent risk factors of MMD and its subtypes.

Independent factors	MMD		Ischemic MMD		Hemorrhagic MMD	
	OR (95% CI)	*p*-Value	OR (95% CI)	*p*-Value	OR (95% CI)	*p*-Value
Age, y	1.015 (0.994–1.038)	0.169	1.016 (0.993–1.039)	0.184	1.014 (0.988–1.040)	0.283
Gender	1.004 (0.627–1.607)	0.987	1.148 (0.705–1.870)	0.579	0.703 (0.390–1.267)	0.241
Heart rate, bpm	1.015 (0.982–1.049)	0.373	1.008 (0.976–1.042)	0.617	1.025 (0.988–1.063)	0.191
SBP, mmHg	1.061 (1.039–1.083)	<0.001 *	1.072 (1.047–1.097)	<0.001 *	1.040 (1.015–1.067)	0.002 *
DBP, mmHg	1.044 (1.016–1.072)	0.002 *	1.052 (1.022–1.084)	0.001 *	1.027 (0.993–1.062)	0.117
BMI, kg/m^2	1.097 (1.030–1.168)	0.004 *	1.133 (1.059–1.213)	<0.001 *	1.022 (0.947–1.102)	0.579
WBC count, 10^9/L	1.433 (1.222–1.680)	<0.001 *	1.527 (1.284–1.816)	<0.001 *	1.268 (1.073–1.499)	0.005 *
LY count, 10^9/L	1.348 (0.908–2.003)	0.139	1.791 (1.142–2.810)	0.011 *	0.869 (0.567–1.332)	0.520
NEUT count, 10^9/L	1.534 (1.255–1.876)	<0.001 *	1.621 (1.307–2.010)	<0.001 *	1.340 (1.103–1.628)	0.003 *
MONO count, 10^9/L	5.628 (0.816–38.821)	0.080	8.682 (1.141–66.063)	0.037 *	2.019 (0.185–22.042)	0.564
PLT count, 10^9/L	1.002 (0.998–1.005)	0.376	1.002 (0.999–1.006)	0.211	1.000 (0.995–1.004)	0.878
ALT, U/L	1.016 (1.001–1.032)	0.036 *	1.020 (1.004–1.037)	0.015 *	1.008 (0.991–1.025)	0.362
ALP, U/L	1.032 (1.017–1.047)	<0.001 *	1.031 (1.015–1.046)	<0.001 *	1.036 (1.018–1.055)	<0.001 *
Glu, mmol/L	1.405 (1.077–1.832)	0.012 *	1.646 (1.209–2.242)	0.002 *	0.935 (0.635–1.377)	0.733
Urea, mmol/L	0.910 (0.792–1.045)	0.183	0.895 (0.758–1.056)	0.190	0.940 (0.830–1.065)	0.331
Cr, μmol/L	0.994 (0.978–1.010)	0.461	0.995 (0.978–1.012)	0.568	0.992 (0.973–1.011)	0.384
TG, mmol/L	1.774 (1.194–2.634)	0.005 *	1.852 (1.224–2.803)	0.004 *	1.499 (0.991–2.268)	0.055
TC, mmol/L	0.626 (0.488–0.804)	<0.001 *	0.514 (0.388–0.682)	<0.001 *	0.887 (0.633–1.243)	0.486
HDL-C, mmol/L	0.080 (0.034–0.188)	<0.001 *	0.059 (0.023–0.149)	<0.001 *	0.141 (0.047–0.424)	<0.001 *
LDL-C, mmol/L	0.657 (0.495–0.873)	0.004 *	0.513 (0.373–0.707)	<0.001 *	1.057 (0.725–1.541)	0.773
ApoA, g/L	0.177 (0.066–0.473)	0.001 *	0.162 (0.058–0.457)	0.001 *	0.142 (0.035–0.578)	0.006 *
ApoB, g/L	1.473 (0.504–4.304)	0.479	0.974 (0.310–3.060)	0.964	3.945 (1.051–14.811)	0.042 *
Hcy, μmol/L	1.075 (1.019–1.134)	0.008 *	1.073 (1.019–1.131)	0.008 *	1.056 (0.994–1.122)	0.080
PNR	0.982 (0.973–0.991)	<0.001 *	0.980 (0.971–0.990)	<0.001 *	0.985 (0.975–0.996)	0.008 *
SII	1.001 (1.000–1.002)	0.030 *	1.001 (1.000–1.001)	0.070	1.001 (1.000–1.002)	0.017 *
MHR	159.576 (14.011–1817.491)	<0.001 *	393.369 (29.479–5249.145)	<0.001 *	19.489 (1.203–315.758)	0.037 *
Neu5Ac, μmol/L	1.395 (1.141–1.706)	0.001 *	1.299 (1.059–1.593)	0.012 *	1.666 (1.294–2.144)	<0.001 *

MMD, moyamoya disease; OR, odds ratio; CI, confidence interval; SBP, systolic blood pressure; DBP, diastolic blood pressure; BMI, body mass index; WBC, white blood cell; LY, lymphocyte; NEUT, neutrophil; MONO, monocyte; PLT, platelet; ALT, alanine transaminase; ALP, alkaline phosphatase; Glu, glucose; Cr, creatinine; TG, triglyceride; TC, total cholesterol; HDL-C, high-density lipoprotein cholesterol; LDL-C, low-density lipoprotein cholesterol; ApoA, apolipoprotein A; ApoB, apolipoprotein B; Hcy, homocysteine; PNR, platelet-to-neutrophil ratio; SII, systemic immune-inflammation index; MHR, monocyte-to-HDL cholesterol ratio; Neu5Ac, N-acetylneuraminic acid. *$p < 0.05$, significant difference.

Table S2. Model collinear statistical analysis of MMD.

Variables	Continuous Neu5Ac		Binary of Neu5Ac		Quartiles of Neu5Ac	
	p-Value	VIF	*p*-Value	VIF	*p*-Value	VIF
Constant	0.077		0.065		0.035*	
Age, y	0.753	1.162	0.627	1.158	0.637	1.159
Gender	0.333	2.080	0.222	2.086	0.251	2.079
SBP, mmHg	<0.001 *	1.727	0.001 *	1.725	0.001 *	1.725
DBP, mmHg	0.707	1.592	0.775	1.590	0.759	1.590
BMI, kg/m^2	0.962	1.239	0.971	1.240	0.918	1.241
PLT count, 10^9/L	0.199	2.301	0.148	2.291	0.191	2.301
ALT, U/L	0.642	1.173	0.689	1.173	0.690	1.173
ALP, U/L	0.125	1.097	0.121	1.097	0.124	1.097
Glu, mmol/L	0.911	1.156	0.885	1.156	0.982	1.156
Urea, mmol/L	0.002 *	1.160	0.003 *	1.158	0.002 *	1.161
Cr, μmol/L	0.023 *	1.891	0.041 *	1.879	0.025 *	1.892
TG, mmol/L	0.021 *	1.365	0.020 *	1.365	0.020 *	1.365
TC, mmol/L	<0.001 *	4.065	<0.001 *	4.055	<0.001 *	4.057
ApoB, g/L	<0.001 *	4.055	<0.001 *	4.035	<0.001 *	4.043
Hcy, μmol/L	0.014 *	1.250	0.019 *	1.249	0.020 *	1.248
PNR	0.001 *	2.492	0.002 *	2.492	0.002 *	2.493
SII	0.850	1.810	0.779	1.808	0.815	1.808
MHR	0.583	1.691	0.646	1.693	0.717	1.696
Neu5Ac	<0.001 *	1.053	0.017 *	1.039	0.001 *	1.062

MMD, moyamoya disease; Neu5Ac, N-acetylneuraminic acid; VIF, variance inflation factor; SBP, systolic blood pressure; DBP, diastolic blood pressure; BMI, body mass index; PLT, platelet; ALT, alanine transaminase; ALP, alkaline phosphatase; Glu, glucose; Cr, creatinine; TG, triglyceride; TC, total cholesterol; ApoB, apolipoprotein B; Hcy, homocysteine; PNR, platelet-to-neutrophil ratio; SII, systemic immune-inflammation index; MHR, monocyte-to-HDL cholesterol ratio. *$p < 0.05$, significant difference.

Table S3. Model collinear statistical analysis of ischemic MMD.

Variables	Continuous Neu5Ac		Binary of Neu5Ac		Quartiles of Neu5Ac	
	p-Value	VIF	*p*-Value	VIF	*p*-Value	VIF
Constant	0.240		0.153		0.168	
Age, y	0.999	1.202	0.916	1.199	0.857	1.198
Gender	0.916	2.409	0.760	2.420	0.820	2.407
SBP, mmHg	<0.001 *	1.759	0.001 *	1.757	0.001 *	1.759
DBP, mmHg	0.593	1.587	0.735	1.576	0.737	1.574
BMI, kg/m^2	0.667	1.262	0.693	1.263	0.705	1.263
PLT count, 10^9/L	0.056	2.612	0.045 *	2.633	0.050	2.616
ALT, U/L	0.971	1.214	0.958	1.213	0.972	1.213
ALP, U/L	0.216	1.104	0.209	1.104	0.213	1.104
Glu, mmol/L	0.397	1.182	0.359	1.181	0.407	1.183
Urea, mmol/L	0.004 *	1.272	0.006 *	1.267	0.004 *	1.270
Cr, μmol/L	0.014 *	2.142	0.028 *	2.117	0.018 *	2.133
TG, mmol/L	0.006 *	1.330	0.007 *	1.331	0.007 *	1.330
TC, mmol/L	<0.001 *	4.203	<0.001 *	4.206	<0.001 *	4.200
ApoB, g/L	<0.001 *	4.220	<0.001 *	4.200	<0.001 *	4.213
Hcy, μmol/L	0.017 *	1.262	0.025 *	1.257	0.021 *	1.259
PNR	0.004 *	2.538	0.003 *	2.539	0.003 *	2.534
SII	0.677	2.112	0.568	2.107	0.593	2.100
MHR	0.610	1.805	0.578	1.808	0.584	1.804
Neu5Ac	0.007 *	1.081	0.148	1.058	0.030 *	1.070

MMD, moyamoya disease; Neu5Ac, N-acetylneuraminic acid; VIF, variance inflation factor; SBP, systolic blood pressure; DBP, diastolic blood pressure; BMI, body mass index; PLT, platelet; ALT, alanine transaminase; ALP, alkaline phosphatase; Glu, glucose; Cr, creatinine; TG, triglyceride; TC, total cholesterol; ApoB, apolipoprotein B; Hcy, homocysteine; PNR, platelet-to-neutrophil ratio; SII, systemic immune-inflammation index; MHR, monocyte-to-HDL cholesterol ratio. *$p < 0.05$, significant difference.

Table S4. Model collinear statistical analysis of hemorrhagic MMD.

Variables	Continuous Neu5Ac		Binary of Neu5Ac		Quartiles of Neu5Ac	
	p-Value	VIF	*p*-Value	VIF	*p*-Value	VIF
Constant	0.793		0.763		0.895	
Age, y	0.384	1.292	0.342	1.291	0.422	1.295
Gender	0.138	1.993	0.083	1.986	0.098	1.987
SBP, mmHg	0.010 *	1.708	0.010 *	1.708	0.009 *	1.708
DBP, mmHg	0.771	1.682	0.594	1.683	0.523	1.685
BMI, kg/m²	0.988	1.317	0.977	1.317	0.987	1.317
PLT count, 10^9/L	0.441	2.387	0.710	2.386	0.663	2.383
ALT, U/L	0.413	1.281	0.314	1.273	0.530	1.297
ALP, U/L	0.005 *	1.211	0.006 *	1.210	0.005 *	1.210
Glu, mmol/L	0.050 *	1.311	0.086	1.314	0.056	1.311
Urea, mmol/L	0.054	1.402	0.091	1.395	0.054	1.402
Cr, µmol/L	0.388	2.039	0.377	2.042	0.353	2.042
TG, mmol/L	0.618	1.931	0.397	1.955	0.434	1.942
TC, mmol/L	0.001 *	3.606	0.001 *	3.586	0.001 *	3.591
ApoB, g/L	<0.001 *	4.169	0.001 *	4.233	<0.001 *	4.187
Hcy, µmol/L	0.160	1.296	0.150	1.298	0.228	1.312
PNR	0.102	2.603	0.159	2.600	0.127	2.600
SII	0.271	2.046	0.317	2.045	0.366	2.045
MHR	0.406	1.723	0.641	1.696	0.671	1.691
Neu5Ac	<0.001 *	1.105	0.001 *	1.099	<0.001 *	1.104

MMD, moyamoya disease; Neu5Ac, N-acetylneuraminic acid; VIF, variance inflation factor; SBP, systolic blood pressure; DBP, diastolic blood pressure; BMI, body mass index; PLT, platelet; ALT, alanine transaminase; ALP, alkaline phosphatase; Glu, glucose; Cr, creatinine; TG, triglyceride; TC, total cholesterol; ApoB, apolipoprotein B; Hcy, homocysteine; PNR, platelet-to-neutrophil ratio; SII, systemic immune-inflammation index; MHR, monocyte-to-HDL cholesterol ratio. *$p < 0.05$, significant difference.

**Table S5. Association between different quartiles of Neu5Ac and the risk of MMD and its subtypes.**							
Neu5Ac	No. of	Crude Model		Model 1		Model 2	
	Events (%)	OR (95% CI)	*P*-Value	OR (95% CI)	*p*-Value	OR (95% CI)	*p*-Value
MMD overall	360 (80.2)						
Quartiles							
Q1 (<3.82)	81 (72.3)	1.0 (Ref.)		1.0 (Ref.)		1.0 (Ref.)	
Q2 (3.82–<4.52)	90 (80.3)	1.566 (0.839–2.920)	0.159	1.430 (0.744–2.747)	0.283	2.038 (0.918–4.525)	0.080
Q3 (4.52–<5.59)	89 (78.7)	1.419 (0.770–2.617)	0.262	1.427 (0.747–2.723)	0.281	1.878 (0.869–4.059)	0.109
Q4 (≥5.59)	100 (89.2)	3.189 (1.540–6.604)	0.002 *	2.884 (1.350–6.159)	0.006 *	4.039 (1.650–9.888)	0.002 *
p for trend		1.372 (1.108–1.700)	0.004 *	1.347 (1.075–1.689)	0.010 *	1.500 (1.143–1.970)	0.004 *
Ischemic MMD	258 (74.3)						
Quartiles							
Q1 (<3.75)	56 (65.1)	1.0 (Ref.)		1.0 (Ref.)		1.0 (Ref.)	
Q2 (3.75–<4.45)	64 (73.5)	1.491 (0.777–2.858)	0.229	1.453 (0.720–2.931)	0.296	2.493 (1.018–6.107)	0.046 *
Q3 (4.45–<5.37)	67 (77.0)	1.795 (0.920–3.500)	0.086	1.831 (0.886–3.783)	0.102	1.917 (0.781–4.705)	0.156
Q4 (≥5.37)	71 (81.6)	2.377 (1.180–4.790)	0.015 *	1.931 (0.914–4.081)	0.085	3.123 (1.232–7.918)	0.016 *
p for trend		1.325 (1.063–1.652)	0.012 *	1.258 (0.991–1.596)	0.060	1.395 (1.041–1.868)	0.026 *
Hemorrhagic MMD	102 (53.4)						
Quartiles							
Q1 (<3.76)	16 (64.0)	1.0 (Ref.)		1.0 (Ref.)		1.0 (Ref.)	
Q2 (3.76–<4.68)	22 (45.8)	1.639 (0.716–3.754)	0.242	1.560 (0.650–3.741)	0.320	2.454 (0.765–7.880)	0.131
Q3 (4.68–<5.66)	28 (58.3)	2.712 (1.180–6.237)	0.019 *	2.960 (1.220–7.178)	0.016 *	2.941 (0.998–8.665)	0.050
Q4 (≥5.66)	36 (75.0)	5.812 (2.389–14.144)	<0.001 *	6.598 (2.588–16.819)	<0.001 *	8.442 (2.703–26.364)	<0.001 *
p for trend		1.776 (1.347–2.342)	<0.001 *	1.872 (1.396–2.512)	<0.001 *	1.917 (1.350–2.722)	<0.001 *

Neu5Ac, N-acetylneuraminic acid; MMD, moyamoya disease; OR, odds ratio; CI, confidence interval. *$p < 0.05$, significant difference.
